# Finger-Actuated Micropump of Constant Flow Rate without Backflow

**DOI:** 10.3390/mi14040881

**Published:** 2023-04-19

**Authors:** NurFarrahain Nadia Ahmad, Nik Nazri Nik Ghazali, Ahmad Taufiq Abdul Rani, Mohammad Hafiz Othman, Chia Ching Kee, Prastika Krisma Jiwanti, Arturo Rodríguez-Gómez, Yew Hoong Wong

**Affiliations:** 1 Department of Mechanical Engineering, Faculty of Engineering, Universiti Malaya, Kuala Lumpur 50603, Federal Territory, Malaysia; 2 School of Mechanical Engineering, Faculty of Engineering, Universiti Teknologi Malaysia, Johor Bahru 81310, Johor, Malaysia; 3 Industrial and Mechanical Design, Faculty of Engineering, German-Malaysian Institute, Jalan Ilmiah, Taman Universiti, Kajang 43000, Selangor, Malaysia; 4 Department of Process & Food Engineering, Faculty of Engineering, Universiti Putra Malaysia, Serdang 43400, Selangor, Malaysia; 5 Centre for Advance Materials and Intelligent Manufacturing, Faculty of Engineering, Built Environment & Information Technology, SEGi University, Petaling Jaya 47810, Selangor, Malaysia; 6 Nanotechnology Engineering, Faculty of Advanced Technology and Multidiscipline, Universitas Airlangga, Surabaya 60115, Indonesia; 7 Instituto de Física, Universidad Nacional Autónoma de México, Circuito de la Investigación Científica s/n, Ciudad Universitaria, A.P. 20-364, Coyoacán, Ciudad de México 04510, Mexico

**Keywords:** microfluidic, finger-actuated micropump, Tesla vale, flow rate, diodicity

## Abstract

This paper presents a finger-actuated micropump with a consistent flow rate and no backflow. The fluid dynamics in interstitial fluid (ISF) extraction microfluidics are studied through analytical, simulation, and experimental methods. Head losses, pressure drop, diodocity, hydrogel swelling, criteria for hydrogel absorption, and consistency flow rate are examined in order to access microfluidic performance. In terms of consistency, the experimental result revealed that after 20 s of duty cycles with full deformation on the flexible diaphragm, the output pressure became uniform and the flow rate remained at nearly constant levels of 2.2 μL/min. The flow rate discrepancy between the experimental and predicted flow rates is around 22%. In terms of diodicity, when the serpentine microchannel and hydrogel-assisted reservoir are added to the microfluidic system integration, the diodicity increases by 2% (Di = 1.48) and 34% (Di = 1.96), respectively, compared to when the Tesla integration (Di = 1.45) is used alone. A visual and experimentally weighted analysis finds no signs of backflow. These significant flow characteristics demonstrate their potential usage in many low-cost and portable microfluidic applications.

## 1. Introduction

Microfluidic devices are promising for diagnostic medical applications due to their ability to control the flow behaviors of micro- or nanoliters of fluid for the detection of analytes and biomarkers related to clinically relevant diseases [1,2,3]. The use of biofluids like urine [4,5,6], saliva [7,8], sweat [9], and tears [10,11], has inherent limitations due to low concentrations, impurities that interfere with multiple factors, and a lack of accuracy when diagnosing disease [12,13]. Dermal ISF is useful for continuous health monitoring, diagnosing diseases, and making personalized treatments due to its richness of exclusive biomarkers [14], ease of access, fact that it does not clot [15,16,17], and near-identicality to blood in terms of biomarker composition and profile trend [16,18,19]. These microfluidic platforms are very attractive for carrying out analysis at the point of care (POC) with distinctive merits, including low volume consumption of extraction biofluid samples, high-speed analysis, efficiency, cost-effective, real-time, and portability without the need for a clinical laboratory [20,21,22,23]. Internet-of-things (IoT)-enabled point-of-care (POC) devices have advanced towards robust features to enable rapid diagnosis and therapy, incorporate remote monitoring and controlling of disease, and provide two-way communication with a central medical facility for interpretation. As a result, it can be fully automated with smart detection and highly efficient display result readout, is easy to use, and is cost-effective [24,25,26,27,28].

Accurate liquid flow control is essential for microfluidics to function as intended; this can be accomplished either actively or passively. Microfluidic systems typically have micropump and microvalve integration. In an active micropump, a constant flow with a controllable flow rate can be controlled by using an external power source. However, they most often deal with high flow rates and have high power consumption, making them less practical in most applications [29]. In passive microfluidics, there is no need for an outside source of energy, and the flow rates depend on the properties of the fluid, the material used, and the design of the microfluidics [30]. Chemical gradients, osmotic pressure, degassed polydimethylsiloxane (PDMS), permeation in PDMS, and capillary forces can all be used to power a passive micropump. However, most passive micro-pumping methods still suffer in terms of controlling and keeping a constant flow rate [31]. Among the aforementioned techniques, a finger-driven approach that is classified under passive control can bring significant advances towards the simplicity, cost effectiveness, and ease of use of microfluidic devices. As the simplest approach, a finger actuation deformable pressure cavity can be used to apply pressure directly or indirectly into microfluidic channels [32,33,34,35,36,37,38,39,40,41,42,43]. Releasing or pushing of the pressure chamber produces a negative or a positive pressure into microfluidic channels. However, the capability to use finger-powered micropumps to perform advanced microfluidic operations system has remained a significant challenge [32]. Although finger actuation is a simple action that anyone can easily accomplish, there would be variation in the individual finger actuation producing different magnitudes of force, leading to user-dependent errors [32,37,44,45]. Thus far, there is very limited research work that has developed a microfluidic-assisted finger-powered micropump. Most of them use valve. For example, Sweet et al. [29] used two fluidic diodes and Ruiz et al. [35] used two ball valves to allow fluidic flows only in the left-to-right direction.

A valve is a critical tool that acts as an open-close switch to manipulate fluid flow direction and flow rate. A valve can either be passive or active. Passive valves have a simpler design and do not require any sort of external power source to operate, while active valves do. It can be further divided into mechanical and non-mechanical based on moving parts. Concern about wear, fatigue, valve blocking, and complexity makes mechanical valves less useful in integrated microfluidic systems, limiting their potential applications. Tesla valve is one of non-mechanical passive valves [46]. With no moving part (or fixed geometry valve) that overcome the drawback of check valve such as wear and fatigue failure, but their output pump efficiency is limited to about 19–27% [47,48,49] (backward flow cannot be blocked completely which is one of the biggest drawbacks in microfluidic system) [50,51]. The Tesla valve is easily miniaturized, resulting in improved operational stability, ascendability, versatility, reliability, and ease of fabrication across a wide range of materials [52]. The Tesla valve has an excellent one-direction flow characteristic that is able to enhance forward flow and prevent reverse flow [53]. However, under low Reynold number condition (Re ≤ 1) in microscale flow, Tesla valve design does not maintain the required diodicity to provide the fully one-direction forward flow [54]. Based on a comprehensive study of existing passive valves at low Reynolds numbers conducted by Fadl et al. [55], and none of them showed a sufficiently high diodicity. To enhance the diodicity, some researcher used microparticle [56] and stimuli hydrogel [57,58]. In order to speed up water absorption for suction, Seo et al. [59] uses a superabsorbent hydrogel contained in a housing with porous fins. The authors reported that this design was able to give a flow rate ≥80 μL/min in an absorption volume of 20 mL. Tesla valve and hydrogel has been studied in literature before but combination of both techniques with serpentine microchannel on backflow minimization in low Reynold number condition has not yet been carried out.

With the aim for a constant flow rate and no backflow in a low-cost microfluidic device, to the best of our knowledge, this is the first finger-actuated microfluidic system that can generate a constant flow rate, and the integration of a Tesla-serpentine-hydrogel reservoir can minimize backflow. The advantages of the intended microfluidic concept are as follows:

1. Easy to fabricate by using the Embedded Scaffold Removing Open Technology (ESCARGOT) method.
2. Controlling the total deformation of the flexible diaphragm until the bottom chamber of a finger-actuated micropump allows for indirect regulation of the volume of fluid moving.
3. A combination of Tesla valves, serpentine microchannels, and hydrogel-assisted reservoirs can increase the reverse flow resistance, in other words, no backflow can be obtained. This can reduce the risk of hygiene and make sensing reading redundant.

### 1.1. Proposed Concept Design

Microneedles (MNs) are a minimally invasive method that can be used to gain access to ISF under the skin with minimal discomfort and physical damage and a low risk of infection [60,61,62,63]. Even though it is known that ISF has useful biomarkers, the extraction process is tedious, which reduces its acceptance in clinical and diagnostic settings. High fluidic resistance within the dermis from extracellular matrix (ECM) causes ISF to be retained by the skin; in contrast to blood, ISF does not flow out instantly due to the presence of net hydrostatical pressure by body circulation effect. As the diffusion rate of ISF during the extraction process is low, an external force is required to collect ISF from the skin [64,65,66]. ISF extraction with MNs also requires a low insertion force, a high insertion success rate, and the ability to reach the desired dermal layer without causing excessive skin damage or bleeding; otherwise, it defeats the initial purpose of being non-invasive. There is still a lot of room for improvement in these platforms, especially in aspects of design, large-scale manufacturing, fluid control, sample handling, integration, and analysis techniques, which are still in the early stages of development.

Miniature, standalone, and user-friendly designs are needed to fulfil liquid control needs such as maintaining the flow rate at a high consistency within the desired rate and sufficient sampling size for a certain period of time. Basically, several general requirements should be taken into consideration in MNs-based ISF extraction POC microfluidic device development [31]:

1. Take note that the extraction rate should not go above 1–2 mL/min to prevent discomfort, pain, distortion, and tearing of the tissue [67,68,69]. Hence, the low extraction rate (from 10 µL/min to a few hundred microliters) is painless [69] and suitable for transdermal application [70,71]. 1 μL/min flow rate is appropriate flow rate for maximizing recovery factor (RF) and obtaining high accuracy measurement in detection biosensor [72]. The recovery factor (RF), which is the ratio of the glucose concentration in the extracted ISF to the fresh ISF in the skin, can be calculated using Equation (1). In cases where the flow rate is less than 1 μL/min, although the recovery factor is greater, a longer time is taken to reach the sensor, and a higher sensitivity of the sensor is required [73]. At high flow rates, the working electrodes of the sensor cannot fully interact with the glucose, making the sensor less accurate at detecting glucose [74,75,76].
2. The ability to maintain a consistent flow rate enables stable sensing signals and data transmission for real-time analysis, judgement, and treatment decision-making.
3. It should be consumed in the smallest possible volume for diagnosis within the desired flow rate range. Continuous monitoring, such as measuring glucose levels in a diabetic patient, is challenging to carry out since it requires a sampling process at a high frequency within a day, and a large sample size is impractical. As long as the sensor is able to analyze the relevant analytes of interest and produce a sensing signal using a minimal sampling size that is applied to the sensing reaction area, it is appropriate for the overall system. Laboratory techniques may require a sample volume of >100 μL for precise analysis and lengthy testing times. However, in practice, most diagnosis devices extract a small volume of 1–10 μL of ISF [17,77] and 0.6–1.1 μL of blood sampling size for biosensors [78], respectively, within a minute [79]. A adequate of ISF sample can produce an accurate and reliable measurement, so a larger volume of sample fluid than is necessary to fully saturate the sensing region’s reaction area is recommended. Incorrect measurements can lead to wrong diagnoses and ineffective treatments.
4. It should have robust performance under various conditions and be economical. High manufacturing costs are a barrier to the widespread use of microfluidic devices since they are required to integrate multiple components to achieve desired results. Single-use devices (disposables) have had an overwhelmingly large impact on society and the economy compared to applications that consider continuous long-term monitoring strategies.
5. No backflow to avoid hygiene or incorrect analysis due to repeated use of the same sample
6. Leakage-free

Here, the microfluidic device is designed and fabricated to be used for POC application and to fulfil the general requirement for single use up to 24 h to extract the ISF from the body for glucose monitoring. The designed microfluidic device has two main components: a finger-powered actuation part and an integrated fluidic network consisting of an MNs array, a tesla valve, a 28-mm length of serpentine microchannel, and a hydrogel-assisted reservoir. The total size of the pump body is 50 mm in diameter and 5 mm in height. Due to the limitations of the stair-stepping effect, microfluidic devices that use 3D-printed sacrificial templates cannot be made with circular microchannels. So, the microchannels, which have a rectangular cross section of 50 μm in height and 100 μm in width, are developed. Volumetric flow rate, Q can be calculated using Equation (2). Single-needle volumetric flow rate, $Q_{\left(n=1\right)}$ of $2.5\;\times \;10^{-14}{\;m}^{3}/s$ (0.0015 μL/min) is based on the extraction of ISF hollow MNs by Samant et al. [64] has been shown to satisfy ISF extraction conditions and, hence, is chosen as the starting point in this work. The inlet is connected to a group of MNs, and the MNs geometry is taken from our previous work [80]. These MNs were designed with a minimal internal bore vortex formation of side-open hollow MNs, which has the benefit of reducing the risk of clogging [81,82,83,84,85], caused by the flow resistance caused by the compression of dense dermal tissue around the MN tips during insertion [86,87]. By using 120 pieces of MNs, the extraction process has a total $Q_{\left(n=120\right)}$ of $3.0\;\times \;10^{-12}{\;m}^{3}/s$ (0.18 μL/min) and the extracted volume is expected to be about 259.2 μL within 24 h. The glucose sensor is allocated at the top inlet, allowing it to collect fresh ISF as it enters the system via microneedles for immediate analysis, expedite the diagnosis process, and reduce the risk of biofouling on the electrochemical sensor by maintaining the fluid flow. The inlet cavity is 250 μm in diameter and 50 μm in height, which gives a cavity volume of 2.45 × 10^−12^ m^3^. Based on the given $Q_{\left(n=120\right)}$, ideally, ISF will take 0.8-s (~0.014 min) to fill up the inlet cavity and reach the sensor for analysis. Assuming the space between the MNs array and the inlet is very small, the volume gap is negligible. The sensor region area will connect with the working electrode of reverse iontophoresis to enhance the extraction process. However, due to Fused Deposition Modeling (FDM) limitation, the prototype microfluidic device design is scaled up 10 times higher from original design and the scale up volumetric intended volumetric, ${*Q}_{\left(n=120\right)}$ is $3.0\;\times \;10^{-11}{\;m}^{3}/s$ (1.8 μL/min).

(1) $$\mathrm{RF}=\frac{C_{\mathrm{glucose},\;\mathrm{Extracted}\;\mathrm{ISF}}}{C_{\mathrm{glucose},\;\mathrm{ISF}}}$$

(2) $$Q=\frac{V}{t}=\frac{\mathrm{Ad}}{t}=A\overset{-}{v}$$

where C is the concentration glucose, Q is the volumetric flow rate, A is the cross-sectional area, d is the distance of fluid moving in units of seconds, and $\overset{-}{v}$ is the mean velocity.

Suction and compression strokes are the two primary actions of a pumping mechanism. The oscillation of the deformation motion caused by changes in internal pressure periodically excited by the finger actuators has a significant impact on the swept volume, or the total amount of fluid in motion [88]. First, finger-driven axial force is applied to the top of a deformable finger actuator to create enough head pressure (positive pressure) to force the air out of the microfluidic. In suction mode, when the finger pressure is removed, the flexible deformable diaphragm returns to its original shape, which generates a negative vacuum pressure inside the fluid chamber, and the fluid is sucked up through MNs array to the sensor that is allocated at the bottom inlet. Due to the Tesla valve’s unique design, which passively encourages one flow direction, more fluid flows forward than in the reverse direction. The hydrogel reservoir is used to immediately trap the used ISF in gel. In the compression mode, vice versa, the positive pressure is generated upon repressing, causing the fluid to flow out simultaneously. Less fluid flows out due to the one-way flow direction design of the Tesla valve structure. The presence of a superabsorbent hydrogel and a serpentine microchannel helps to enhance the diodicity under low Reynolds numbers. Once the ISF is absorbed by the hydrogel in the reservoir, there will be almost none outflow fluid accessible. The serpentine microchannel design helps slow down any outflow. Hydrogel reservoirs are designed with built-in air vents to get rid of trapped air and make fluid flow easier. In order to allow the hydrogel to be poured into the reservoir cavity from the outside, an open-closed outlet port is designed. Using a sequential push-and-release motion, fluids flow through the system [32]. At these levels, the fluid motion ideally flows in a laminar condition without chaotic changes in pressure and flow velocity (turbulence) [89].

**Figure 1:**
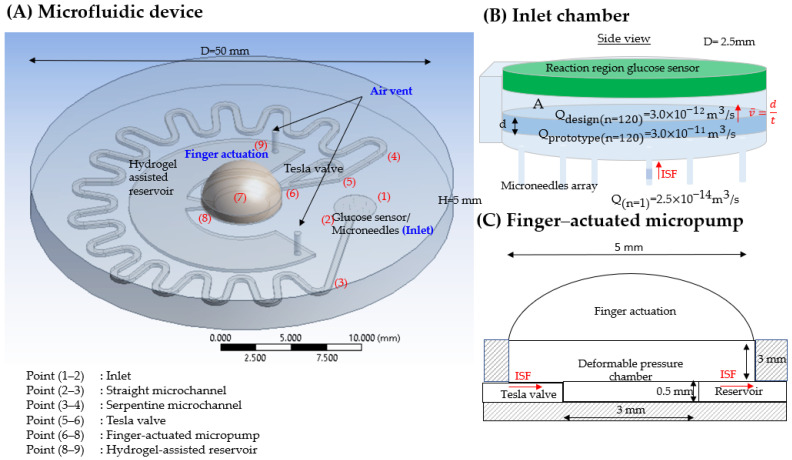
Schematic illustration of the microfluidic device: (**A**) General overview; (**B**) Inlet connected with sensor and microneedle array; (**C**) Finger actuated micropump. (Points (1)–(9) indicated the point-to-point components in the microfluidic system, and the red arrow indicated ISF moving flow).

Figure 1 illustrates the basic concept design of the microfluidic device and its geometry described in Table 1.

### 1.2. Theoretical Analysis

#### 1.2.1. Consistent Flow Rate Analysis

This microfluidic system employs a finger-activated micropump that is fully mechanically deformable by axial force to reach the bottom chamber [32]. In the ideal case, the pressure cannot rise any further once the pressure chamber has deformed to the bottom substrate [32], and this will minimize the effects of user error in the indirect control of pressure and fluid flow [37]. The dome-shaped diaphragm is stronger and has higher rigidity than a flat diaphragm, and it can release the diaphragm’s residual stress by changing its shape as a function of volume. Therefore, a dome diaphragm is capable of producing more force, a higher restoring force of pressure [90], and able to operate at a much higher frequency (~1000 times higher) than a flat diaphragm [91]. The flow rate is determined by chamber geometry and remains constant when the displacement induced by finger actuation is constant. Assuming that the system is under isothermal condition (temperature is constant) and there is no leakage in the pressing-release process [32], the amount of fluid increases as a function of pressing-releasing applied the pump chamber [40], regardless of the displacement actuation or the time interval between pushes [37]. We developed an analytical equation using experimental data from a finger-powered microfluidics device [32]. The average pressure produced by a human finger is ≈4500 Pa [36]. Literature reports vary widely on the ISF’s viscosity, with values as low as $1.2–1.5\;\times \;10^{-3}\;\mathrm{Pa}.s$ [92] and as high as $3.5\;\times \;10^{-3}\mathrm{Pa}.s$ [93]; a mean value of 2.5 × 10^−3^ Pa.s is used [36]. ISF has a density of 1000 kg.m^−3^ [93] and a surface tension of 70 dyn/cm (0.07 N/m) [94].

Let consider a general mathematic cosine function such as f(x) = A cos (B x + C) + D, where A = 2250, B = 2π/5, C = 0, D = 2250. The value A represent a maximum amplitude of the chamber. The value B is the number of cycles to complete interval from 0 to 2π (360°) and the period is $\frac{2\pi }{B}$. The value C is phase shift (positive value mean shifting to the left). The value D is the vertical shift from center line. The following pressure-time function can be derived for pressing at time intervals of 5 s, as defined in Equation (3)

(3) $$P\left(t\right)=2250\cos\left(\frac{2\pi }{5}t\right)+2250$$

where t and P are time in units of seconds and pressure, respectively. To study the effect of the force input by pressing with fingers, we modified the function of Equation (3) into a version Equation (4) when pressing 5 times faster, which means that the pressing interval is in every second.

(4) $$P\left(t\right)=2250\cos\left(2\pi t\right)+2250$$

**Figure 2:**
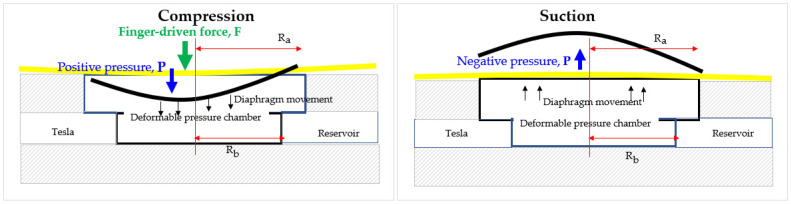
Schematic diagram of compression and suction mode in finger-actuated micropump.

The chamber cavity has a considerably smaller diameter than the finger dome-shape actuator, which reduces the liquid’s compressibility and causes less positive pressure to force the working fluid out [95]. The force diagram of the finger-actuated micropump is shown in Figure 2. $R_{a}$ and $R_{b}$ denote the radius of the finger-actuator and deformable pressure chamber, respectively. F is the finger-driven force, and P is the pressure inside the cavity.

#### 1.2.2. Backflow Analysis

**Figure 3:**
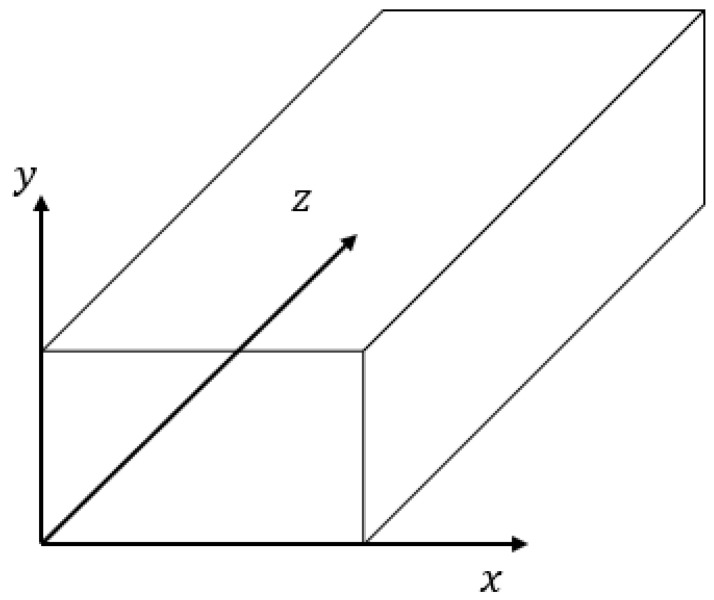
Coordinate system and cross-section of a rectangular channel.

Figure 3 depicts a cross section of a rectangular microchannel (Aspect ratio, β= height/width = 0.5/1 = 0.5). Reasonable assumptions for fluid dynamics in this microfluidic device are outlined as below:

1. ISF used as working fluid is Newtonian fluid
2. The fluid flow is laminar (Re < 2300) [89], incompressible fluid flow $(\nabla \overset{⃑}{V\;}=0;\;\frac{\partial u}{\partial x}+\frac{\partial v}{\partial y}+\frac{\partial w}{\partial z}=0)$ in which the fluid properties, such as its density and viscosity, are constant; steady flow $(\frac{\partial \rho }{\partial t}=0)$ in which density does not change as a function of time
3. There will be no leakage or additional fluid in the system; in other words, the volumetric mass flow rate will be the same everywhere in the system (Qin=Qout)
4. Isothermal (Temperature = constant)
5. Fully developed $\left(\frac{\mathrm{dw}}{\mathrm{dt}}=0\right)$. A constant velocity profile is maintained by the flow when the pressure gradient and shear forces are equalized. The z-axis pressure gradient remains constant [96].
6. Velocity along z-axis is function of x and y. (u=0, v=0, w ≠0)
7. Fluid flow moving horizontally, thus gravity effect is negligible (g = 0)
8. No-slip condition is applied on the walls [97,98]

Continuity and Navier-Stokes equation for steady, incompressible Newtonian fluid are used to simulate the fluidic motion in the microchannels expressed in Equations (5) and (6)

(5) $$\frac{\partial \rho }{\partial t}+\nabla .(\rho .\overset{⃑}{V})=0\;\frac{\partial \rho }{\partial t}+\frac{\partial (\rho u)}{\partial x}+\frac{\partial (\rho v)}{\partial y}+\frac{\partial (\rho w)}{\partial z}=0$$

(6) $$x\;\mathrm{direction}:\;\rho \left(\frac{\partial u}{\partial t}+u\frac{\partial u}{\partial x}+v\frac{\partial u}{\partial y}+w\frac{\partial u}{\partial z}\right)=-\frac{\partial p}{\partial x}+\mu \left(\frac{\partial^{2}u}{{\partial x}^{2}}+\frac{\partial^{2}u}{{\partial y}^{2}}+\frac{\partial^{2}u}{{\partial z}^{2}}\right)+\rho g_{x}\;y\;\mathrm{direction}:\;\rho \left(\frac{\partial v}{\partial t}+u\frac{\partial v}{\partial x}+v\frac{\partial v}{\partial y}+w\frac{\partial v}{\partial z}\right)=-\frac{\partial p}{\partial y}+\mu \left(\frac{\partial^{2}v}{{\partial x}^{2}}+\frac{\partial^{2}v}{{\partial y}^{2}}+\frac{\partial^{2}v}{{\partial z}^{2}}\right)+\rho g_{y}\;z\;\mathrm{direction}:\;\rho \left(\frac{\partial w}{\partial t}+u\frac{\partial w}{\partial x}+v\frac{\partial w}{\partial y}+w\frac{\partial w}{\partial z}\right)=\;\;\;\;\;\;\;\;\;\;\;\;\;\;\;\;\;\;\;\;\;\;\;\;\;\;\;\;\;\;\;\;\;\;\;\;\;\;\;\;\;\;\;\;\;\;\;\;\;\;\;\;\;\;\;\;\;\;-\frac{\partial p}{\partial z}+\mu \left(\frac{\partial^{2}w}{{\partial x}^{2}}+\frac{\partial^{2}w}{{\partial y}^{2}}+\frac{\partial^{2}w}{{\partial z}^{2}}\right)+\rho g_{z}$$

The simplified Navier-Stoke based on abovementioned assumption expressed in Equation (7).

(7) $$\frac{\partial^{2}w}{{\partial x}^{2}}+\frac{\partial^{2}w}{{\partial y}^{2}}=\frac{1}{\mu }.\frac{\partial p}{\partial z}$$

As the flow is fully developed, the boundary condition of V = 0 at x = 0, x = 1; and V = 0 at y = 0, Y = AR = 0.5). The Reynold number (Re) which depends on hydraulic diameter (D_h_) for rectangular microchannel. Re, Dh, and Le described in Equations (8)–(10). The Darcy friction factor (f) for laminar flow [98,99] with a function of pressure drop expressed in Equation (11).

(8) Re=QDμA=Acv−DμA=ρv−Lμ=ρv−Dhμ

(9) $$D_{h}=\frac{4A_{c}}{p},D_{h,\mathrm{rect}.}=\frac{2\mathrm{wh}}{w+h}$$

(10) Le=0.05Dh.Re

(11) $$f=\frac{2D_{h}\Delta p}{\rho L{\overset{-}{v}}^{2}}$$

where ρ is the density, μ is the dynamic viscosity, Ac is the cross-sectional area of the microchannels and p is the wetted-perimeter. L and D are the length and diameter of the microchannel, respectively. ∆p is calculated where the flow is fully developed after the entrance length. The entrance length, defined as the distance where the maximum velocity reaches 99% times the corresponding fully developed value. So, it’s crucial to determine the entrance length as prerequisite [98].

The Extended Bernoulli equation represent modified version of Bernouli principle that take into account gains and losses of the head in unit meter, considered the energy equation for incompressible and steady flow between two locations expressed in Equation (12).

(12) $$\frac{P_{1}}{\rho g}+\frac{v_{1}^{2}}{2g}+Z_{1}+h_{P}=\frac{P_{2}}{\rho g}+\frac{v_{2}^{2}}{2g}+Z_{2}+h_{L}$$

whereas $\frac{P}{\rho g}$ represents the work done, $\frac{v^{2}}{2g}$ represents the kinetic energy, and Z is the elevation of the potential energy. $P_{1}$ and $P_{2}$ are the static pressure at the inlet and outlet respectively, v is the velocity, and g is the gravitational acceleration. $h_{p}$ is the head added by pump while $h_{L}$ is the total head losses. The overall head loss of the system might come from two aspects: (1) Major friction loss can be caused by viscous effect from microchannel length and roughness on the inner surface in straight channel and, (2) Minor local loss can be caused by changes in the geometric shape channel components which incur sudden expansion/contraction. The Darcy-Weisbach formula specifies the following Equations (13) and (14) for determining the magnitude of the major loss ($h_{f}$) and the minor loss ($h_{s}$). The total overall head loss is expressed as per Equation (15).

(13) $$h_{f}=f\frac{L}{D_{h}}\frac{V^{2}}{2g}$$

(14) $$h_{s}=K_{L}\frac{V^{2}}{2g}$$

(15) $$h_{L}=h_{f}+h_{s}=\frac{V^{2}}{2g}\left(\sum \frac{\mathrm{fL}}{D_{h}}+\sum K_{L}\right)$$

where f is the Darcy–Weisbach friction factor, which is a dimensionless quantity to describe frictional losses in internal flow and can be obtained from Moody diagrams. K_L_ is the local loss coefficient, which can be obtained according to the local loss coefficient table and V is average velocity.

The total pressure drops ($\Delta P_{T})$ is sum of the major and minor pressure losses $\left(\Delta P_{T}={\Delta P}_{\mathrm{major}\;\mathrm{losses}}+\Delta P_{\mathrm{minor}\;\mathrm{losses}}\right)$. The major component of the pressure drops ($\Delta P_{\mathrm{major}}$) is happens in the centre of the microchannels due to friction within the channel, which is contributed by two regions: the entrance region when flow is under developing conditions, and the fully developed region afterward, which can be obtained in the remaining length of the channel. The minor losses come from component that involving geometry change such as expansion, contraction, T-junction and bend. Major and minor pressure losses are expressed in Equations (16) and (17)

(16) $$\Delta p_{\mathrm{major}\;\mathrm{losses}}=f\frac{L}{D_{h}}\frac{\rho V^{2}}{2}$$

(17) $$\Delta p_{\mathrm{minor}\;\mathrm{losses}}=K_{L}\frac{{\rho V}^{2}}{2}$$

**Figure 4:**
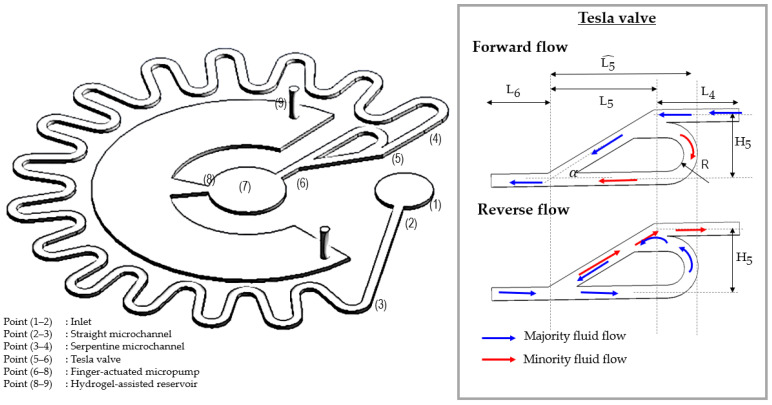
Schematic for microfluidic network.

Here, the microfluidic device is divided into six components for easy understanding of their head losses (illustrated in Figure 4). The first components (Point 1–2) is inlet entrance that involve expansion and contraction which from microneedles array or inlet entrance channel to inlet outflow. The second component (Point 2–3) is straight rectangular channel while third component (Point 3–4) is serpentine channel which designed aids in the slowing of the flow consist 29 pieces of 180° return bend ($K_{\left(180{}^{\circ}\;\mathrm{bend}\right)}$) and L denotes the straight length of the channel. The fourth component is the Tesla valve (Point 4–6) which relies solely on inertial forces and the resulting viscous losses. It consists of a straight channel and a bowed channel, both of which are asymmetrical to accommodate the two flow directions. This asymmetry is constructed in such a way to let the fluid flow relatively easily in one direction, namely the forward direction, whereas the fluid encounters a significantly greater flow resistance to restrict flow in the reverse direction, hereinafter referred to as the backward direction. In the forward flow case, it will enter the straight channel unimpeded, avoid the all-winged path channel, and travel in a relatively straight line to the exit port, allowing the fluid to accelerate due to the pressure flow effect. However, in the case of reverse flow, it loses a lot of energy as it travels up and down each passage and has the most devastating effect at the intersection junction. Therefore, the driving pressure required to reverse the flow is much higher than that required to move the fluid forward [100,101,102]. In addition, intense mixing is produced by increased lateral deflections of the streams at the intersection junction. The incoming fluid bounces off the internal structures, with the redirections being only slight in passing baffles but quickly growing downstream and rerouting after multiple interactions, which eventually turn into the suspension flow abeyance state [103]. As a result, a diode effect occurs. The fifth and sixth pressure losses occur at pump chamber (Point 6–8) and diffuser at reservoir entrance designed with an expanding cross section to let the flow in the expansion direction easily, which requires substantially less driving pressure than flow in the opposite direction and outlet (Point 8–9) respectively due to the sudden expansion and contraction. The reservoir facilitates with hydrogel generates negative pressure by the absorption of hydrogel in the chamber, which aids in the continuous movement of fluid out of the chamber and the prevention of backflow.

Head loss in forward and reverse flow for above components listed in Equations (18)–(23) as follows:

(18) $$h_{L\left(1\to 2\right)}=K_{e(1)}\frac{{V_{1}}^{2}}{2g}+K_{c(2)}\frac{{V_{2}}^{2}}{2g}\;h_{L\left(1\leftarrow 2\right)}{=K}_{e(2)}\frac{{V_{2}}^{2}}{2g}+K_{c(1)}\frac{{V_{1}}^{2}}{2g}$$

(19) $$h_{L(2\to 3)}=f_{3}\frac{L_{(2\to 3)}}{D_{h}}\frac{{V_{(2\to 3)}}^{2}}{2g}\;h_{L(2\leftarrow 3)}={\;f}_{3}\frac{L_{(2\leftarrow 3)}}{D_{h}}\frac{{V_{(2\leftarrow 3)}}^{2}}{2g}$$

(20) $$h_{L(3\to 4)}={29K}_{L}\frac{{V_{(3\to 4)}}^{2}}{2g}+f\frac{L_{(3\to 4)}}{D_{h}}\frac{{V_{(3\to 4)}}^{2}}{2g}\;h_{L(3\leftarrow 4)}={\;29K}_{L}\frac{{V_{(3\leftarrow 4)}}^{2}}{2g}+f\frac{L_{(3\leftarrow 4)}}{D_{h}}\frac{{V_{(3\leftarrow 4)}}^{2}}{2g}$$

(21) $$h_{L\left(4\to 6\right)}={\;f}_{4}\frac{L_{4}}{D_{h}}\frac{{V_{4}}^{2}}{2g}+f_{5}\frac{H}{D_{h}\sin\alpha }\frac{{V_{5}}^{2}}{2g}+{\;f}_{6}\frac{L_{6}}{D_{h}}\frac{{V_{6}}^{2}}{2g}+\hat{f_{5}}\frac{R\pi +\hat{L_{5}}-R}{D_{h}}\frac{{\hat{V_{5}}}^{2}}{2g}+K_{L(4)}\frac{{V_{4}}^{2}}{2g}+{\;K}_{L(5)}\frac{{V_{5}}^{2}}{2g}\;h_{L\left(4⟵6\right)}=f_{4}\frac{L_{4}}{D_{h}}\frac{{V_{4}}^{2}}{2g}+f_{5}\frac{H}{D_{h}\sin\alpha }\frac{{V_{5}}^{2}}{2g}+f_{6}\frac{L_{6}}{D_{h}}\frac{{V_{6}}^{2}}{2g}+\hat{f_{5}}\frac{R\pi +\hat{L_{5}}-R+\frac{H-h}{\sin\alpha }}{D_{h}}\frac{{\hat{V_{5}}}^{2}}{2g}+K_{L(4)}\frac{{V_{4}}^{2}}{2g}+K_{L(5)}\frac{{V_{5}}^{2}}{2g}$$

(22) $$h_{L\left(6\to 8\right)}{=K}_{e(7)}\frac{{V_{7}}^{2}}{2g}+K_{c(8)}\frac{{V_{8}}^{2}}{2g}\;h_{L\left(6\leftarrow 8\right)}{=K}_{e(8)}\frac{{V_{8}}^{2}}{2g}+K_{c(7)}\frac{{V_{7}}^{2}}{2g}$$

(23) $$h_{L\left(8\to 9\right)}=K_{e(8)}\frac{{V_{8}}^{2}}{2g}+K_{c(9)}\frac{{V_{9}}^{2}}{2g}\;h_{L\left(8\leftarrow 9\right)}={\;K}_{e(9)}\frac{{V_{9}}^{2}}{2g}+K_{c(8)}\frac{{V_{8}}^{2}}{2g}$$

where contraction loss coefficient ($K_{c}$) expansion loss coefficient ($K_{e}$). K_c_ and K_e_ can be obtained from $K_{e\;\mathrm{or}\;c}={(1-\frac{A_{1}}{A_{2}})}^{2}$. The pressure drop is the difference in total pressure between the two points. Diodicity (Di) is a metric used to assess the performance of a microfluidic device in both forward and reverse liquid flow conditions, which is also called the ratio of flow resistances. Di can be calculated by Equation (24)

(24) $$Di=\frac{\Delta P_{r}}{\Delta P_{f}}$$

where, $\Delta P_{r}$ and $\Delta P_{f}$ represent pressure drop between inlet and outlet when fluid flows in reverse and forward direction, respectively. The larger Di is, the better performance is [104]. The total energy of the fluid is preserved according to the law of conservation of energy. Under the assumption that the energy generated by the finger remains constant, in order to sustain the conservation energy Bernoulli equation, added head of pump requirement should be calculated based on given volumetric flow rate to overcome total of head loss for above six components. Using Equation (25), we can determine the required pump power (Po).

(25) Po=ρghQ

## 2. Materials and Methods

### 2.1. Material

Acrylonitrile Butadiene Styrene (ABS) filament for 3D printing was purchased from a local supplier (3DGadgets, Kuala Lumpur, Malaysia). Potassium polyacrylate based hydrogel with a safe and non-toxic formulation was purchased from Lazada (Qingdao, China) for the reservoir. Slygard 184 clear liquid silicone Polydimethylsiloxane (PDMS) was purchased from Dow Corning (Midland, MI, USA) for molding use. The saline composition was designed to mimic ISF as closely as possible by buffering with sodium hydroxide (NaOH) and/or hydrochloric acid (HCl) to meet a pH value of 7.4 [105] for experimental.

### 2.2. Simulation and Their Boundary Condition

For the simulation, ISF is used as the working fluid, and Fluent ANSYS 2019 R3 software is used to simulate the flow characteristics and validate the working principle [106]. Inlet velocity is calculated and set upon simulation [97]. In the forward flow case, the pressure is set to be 2250 Pa [36] at the inlet based on the finger actuation pump and 0 Pa at the outlet [51]. In reverse flow cases, the setting is reversed. The wall roughness is set to 0.05 mm in accordance with the consistent 3D printing layer employed in the fabrication of the microfluidic channel sacrifice mold [51]. The head losses and pressure drop for forward/reverse flow are evaluated. The normalized friction constant C* as function of Reynold number, expressed by Equation (26) is used to calculate the discrepancy between the experimental and predicted values of the friction coefficient in the laminar region [96,98,107]

(26) C*=f.Reexperimentalf.Repredicted

### 2.3. Swelling Ratio Profile, Absorption Rate and Water Retention of Hydrogel

To understand the hydrogel’s performance as a reservoir and diodicity enhancement, the swelling ratio profile, absorption rate, and water retention are investigated. It is possible to obtain the profile of the swelling ratio as a function of time by performing the free-absorbency capacity test in four different ways: the teabag method, the sieve method, the filtration method, and the sieve filtration method [108]. Ultra-fine fiber nonwoven fabrics of 9 cm × 7.5 cm teabag are used in this work. At specific time intervals, 0.2 g of dried hydrogel samples are immersed in 100 mL of saline solution (pH 7.4, 37 °C). After the time is up, any remaining liquid on the surface of the hydrogel is taken off with filter paper that can soak up water [109]. After the hydrogels have equilibrated, they are placed in petri dishes at room temperature and weighed again using an analytical balance at certain time intervals until they have reached their final saturated weight. Equation (27) is used to calculate the swelling ratio (SR) of the hydrogel [110,111].

(27) $$\mathrm{SR}=\frac{\left(w_{w}-w_{d}\right)}{w_{d}}\times \;100\%$$

where $w_{d}$ and $w_{w}$ are the initial dried-weight and the wet-weight of hydrogels at predetermined time, respectively. The swelling values obtained from the measurements are compared with a Voigt model [112] as described by Equation (28)

(28) $$S_{t,\mathrm{Voigt}\;\mathrm{model}}=S_{e}(1-e^{-\frac{t}{r}})$$

where t is the time it takes to swell, $S_{t,}$ is the swelling capacity at a certain time, and $S_{e}$ is the swelling capacity to reach equilibrium in an infinite amount of time, or, in other words, the maximum water-holding capacity. The r is donated by the time required to reach 63% of the equilibrium swelling [108]. The swelling rate of hydrogel were calculated using Equation (29) [113]

(29) $$v_{\left(\mathrm{SR}\right)}=\frac{w_{t2}-w_{t1}}{w_{d}(t_{2}-t_{1})}$$

where $t_{1}$ and $t_{2}$ are the average swelling times, $w_{t1}$ and $w_{t2}$ are the weights of the wet-sample at predetermined time, and $w_{d}$ is the weight of the dried hydrogels. Their reservoir flow characteristic is evaluated by varying diffuser geometry and reservoir entrance quantity. However, the hydrogel swelling behavior is limited by space, the actual collected fluid sample in the chamber may be less, and the flow rate absorption may be differed. This would be confirmed during experimental.

### 2.4. Fabrication of Microfluidic Device

With such a straightforward fabrication process, the ESCARGOT method, which has a two-step fabrication process, is used for fabrication. The two-step fabrication process: the first step is the printing of sacrificial templates via FDM and the second step is to dissolve the printed template in cured PDMS using acetone to create an empty cavity for a microfluidic channel. In this study, sacrificial templates are made from ABS. All the structures are created in Ansys and converted to stl. files by Autodesk Mesh software. They are sliced by CURA software and transferred as G-codes to the 3D printer. A low-cost and commercially available FDM 3D desktop printer is used to extrude 1.75 mm of ABS plastic with the aid of a 0.4 mm nozzle. The extruded printed structure is cooled down after printing and solidified on the printing bed to form a microchannel mold [114]. The printer creates parts layer by layer until the desired object is created. Support printing is required for this model to prevent part deformation during the printing process and secure the part so it can be attached to the main body of the printed model. The support printing is carefully and slowly removed using a blade, as its removal process could cause a partial break [115]. However, due to its miniature size, partial breaks occur, especially at the joining part. For those parts broken during support printing removal, they can be repeatedly melted down using acetone or water and attached back manually. The remaining acetone on the printed ABS molds is removed before the PDMS curing process to avoid losing the microstructure’s fundamental shape.

The printed molds were precisely placed into a plastic petri dish container without touching its wall. The ratio of Sylgard 184 base to curing agent is 10:1 [114]. The mixture is manually stirred with a glass rod, and degassing is carried out in a desiccator under vacuum to eliminate the potential of the bubbles from the mixing process in order to reduce microchannel structure defects [116,117]. Basically, they can be cured overnight at room temperature [118], or 60 °C for 2 h [114], or 80 °C for 50 min [119]. The effect of temperature on the PDMS curing condition has been studied in detail by Kalpattu et al. [120]. In this work, curing at room temperature is preferable. Despite the slow curing effect, previous studies show that curing at room temperature results in a very low shrinkage rate of 0.3% compared to curing at high temperatures (1.4% shrinkage at 60 °C and 1.6% shrinkage at 80 °C). Subsequently, they are immersed in the PDMS-inert solvent, which is acetone, leaving an empty cavity inside the PDMS [121]. They ultrasonically vibrated until all the ABS printed mold is dissolved, then rinsed it in deionized water and dried it with a flow of compressed filtered air [114]. With a syringe, acetone is also injected into the microchannels to get rid of the remaining printed mold and make it easier for the fluid to flow in. Even with the ESCARGOT process, 3D printing will always result in a rough surface due to the stair-stepping effect [122]. However, the surface roughness for 3D printed microchannels is negligible, as previous studies reported the surface roughness for FDM printed microchannels is around a few tens of nanometers [115]. Even though it has no discernible effect on fluid flow, the surface roughness does increase flow resistance [123]. A typical characterization test is carried out for 3D-printed mold and PDMS microchannels. In order to identify the optimum window parameters used for printing, three main processing parameters that influence structural integrity and dimensional accuracy are studied. The parameters involved extrusion temperature (230 to 250 °C with an interval of 5 °C), bed temperature (80 to 100 °C with an interval of 5 °C), and print speed (45 to 65 mm/min with an interval of 5 mm/min). To compare the printed molds with the designed 3D model, the width and height of the rectangular microchannel is measured [119]. Cpk is calculated using 32 readings for each condition.

To verify the fluid flow condition in cavity microchannels, the color-dyed water is injected at flow rates ranging from 0.1 μL/min to 1 mL/min at the input port via a syringe pump. Color-dyed water is used in the pre-functional test to improve visibility of any probable leakage [117]. 

### 2.5. Experimental Microfluidic System Versatility Confirmation

**Figure 5:**
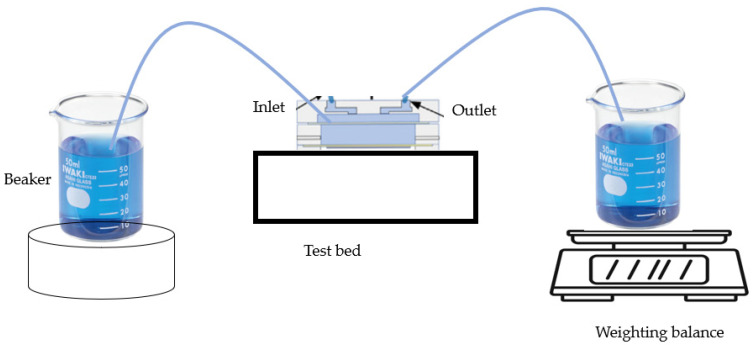
Flow testing system.

The flow testing device is shown in Figure 5. For the flow test, the inlet is connected to a supply beaker, and the outlet is connected to a beaker that placed on a weighting balance. The inlet is connected to a breaker that contained color-dyed liquid throughout this test. To ensure the accuracy of measurement data, 32 readings are taken and the average value is calculated. To demonstrate consistency, ISF mimic sample solution [32] is added to a beaker connected to the inlet and the outlet is connected to the weighting balance. The consistency of flow rate in various interval duty cycles (0.5, 5, 10, and 20 s) is monitored.

## 3. Result and Discussion

### 3.1. Head Loss, Pressure Drop and Diodicity Analysis

**Figure 6:**
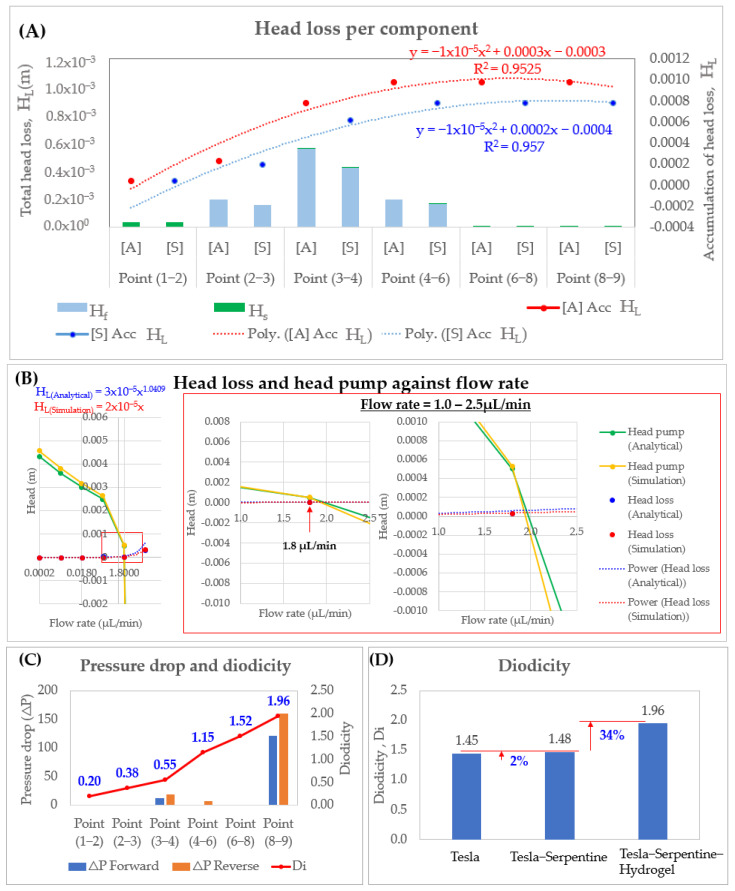
(**A**) Total head losses for each component in microfluidic system. (**B**) Head losses and head pump required against flow rate. **(C**) Point-to-point pressure drop and diodicity. (**D**) Diodicity comparison of Tesla valve, Tesla Serpentine, and Tesla Serpentine-Hydrogel integration in a microfluidic device.

The total head losses for six components (Points 1–9) in this specially developed microfluidic system are shown in Figure 6A. The maximum friction loss is at Points 3–4 of this graph (Serpentine channel). While other components have local losses, only Point 2–3 (Straight Rectangular Channel) and Point 4–6 (Tesla) displayed comparable significant friction loss. When distance and component basis rose, accumulation head losses also increased. No significant difference is found on the head loss value in simulation and analytical. Figure 6B represents the profile of head loss and head pump in the microfluidic system against flow rate (0.000018–18 μL/min). The intersect point between the pump and head loss curves is 0.105–0.115 mm which is well fitted with our design pump location. Diodicity increased in increasing location from the inlet point (Figure 6C). A microfluidic system with tesla alone can produce a diodicity of Di = 1.45. An increase of 2% (Di = 1.48) and 34% (Di = 1.96) in diodicity are observed in the microfluidic system with the addition of the serpentine microchannel and hydrogel, respectively (Figure 6D). This showed that the combination of a tesla, serpentine microchannel, and hydrogel can reduce the backflow in the microfluidic system.

**Figure 7:**
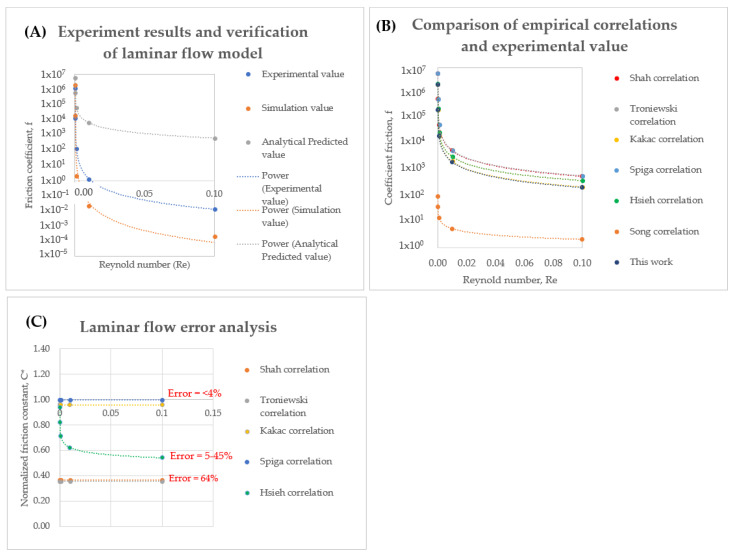
Comparison of friction coefficient (**A**) Experimental, simulation and analytical value in this work (**B**) Comparison of empirical correlation and experimental value (**C**) Laminar flow error analysis.

After checking the performance of head losses, pressure drop, and diodicity, a correlation study is performed to check the error of the gap. The average velocity on a straight rectangular channel is obtained from experimental and simulation used to calculate the friction coefficient under various Re. The comparison result of friction coefficient for experimental, simulation and predicted value (f = 64/Re) [98,124] are shown in Figure 7A. The f value for rectangular channel for experimental is within the analytical predicted and simulation data under Re ranged from 0 to 0.01. However, it could be seen that prediction has a significant gap in this range with 2–20% error. Correlation studies are carried out on eight previous papers with high credibility in order to study on the flow characteristics of a rectangular channel. The f value from previous studies established formula (Equations (30)–(37)) in Table 2 and our experimental data are compared as shown in Figure 7B. In the laminar region, Spiga, and Kakac correlation fitted well (~4% error) with ours (Figure 7C).

### 3.2. Hydrogel-Assisted Reservoir for Interstitial Fluid Collector and Backflow Reduction

**Figure 8:**
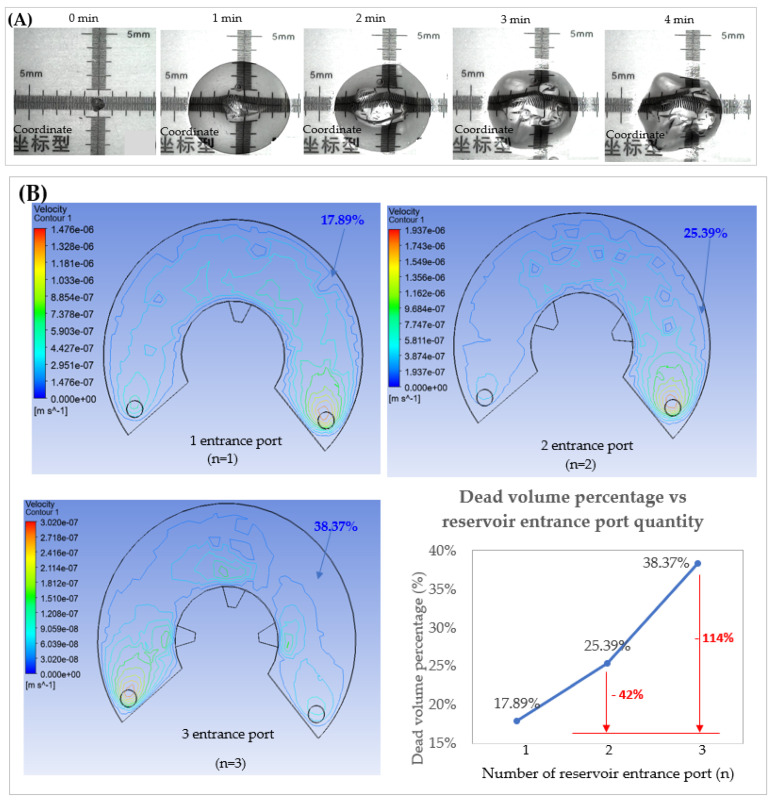

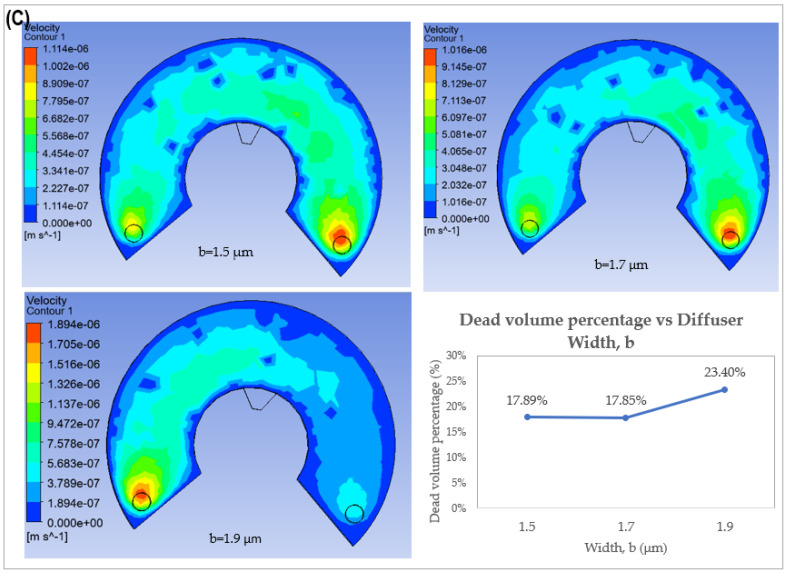
(**A**) Swelling process of one-unit hydrogel after applied single droplet water over time (**B**) Simulated result of storage chamber design optimization by varying (**B**) quantity of reservoir entrance port and (**C**) diffuser width.

The swelling ratio, profile, rate, and saline solution retention capacity of hydrogel at room temperature are investigated to demonstrate its effectiveness as a reservoir and to prevent fluid from moving in the reverse direction. A well-distinguished crystal-like shape is observed in the single bead of hydrogel during the swelling process. The scale on the ruler is displayed in millimeters (mm) to accurately reflect the actual size of the superabsorbent hydrogel utilized in this study. According to data from 10 measurements, a single hydrogel bead takes 4 min to reach after a droplet of saline is applied (SR_(n=1)_ = 65 ± 3). The relative SR in a droplet of saline solution for the 4-min experimental value fits the trend of the Voigt model. Based on a single hydrogel bead, it is also important to note that the bead swelled, indicating absorption once it came into contact with the solution. There were three distinct phases to the absorption behavior: in the first phase (1 min), the amount of water absorbed increased rapidly; in the second phase (2–4 min), absorption slowed down to a moderate speed; and in the third phase (4 min), absorption reached an almost steady state with no further increases in water absorbency (Figure 8A). To examine the swelling performance of a larger quantity of hydrogel, the hydrogel sample is immersed in saline solution via a teabag. After being submerged in saline solution until equilibrium swelling is achieved, the weight of the hydrogel sample (0.22 g) increased to 31.95 g, which is equivalent to a SR of 144 g/g. The obtained SR within the other water-absorbent hydrogel from past studies (80–5520 g/g) [131,132,133,134,135,136]. This is due to the fact that the superabsorbent hydrogel possesses multiple hydrophilic macromolecules and is composed of crosslinked networks that acquire coil conformation in their dry state, but they have the capability to absorb water up to 500 times their own weight [137] and significantly expand their structure to a large size once they are exposed to water or an aqueous medium [138]. In a 20 mL saline, the absorption rate is 83.3 μL/min. The obtained flow rate for this work is almost identical to the flow rate obtained in a previous study by Seo et al. [59] (~80 μL/min and an absorption volume of ~20 mL).

To achieve a maximum static storage area with rapid absorption of ~83 μL/min and a swelling rate of more than 5000 times, the hydrogel-assisted reservoir design and their geometry need to be taken into consideration. This reservoir serves two purposes: to store diagnostic waste samples and to quickly trap fluid in their matrix to keep fluid flow going and keep it from going backward. Based on the given and $Q_{\left(n=120\right)}$ and ${*Q}_{\left(n=120\right)}$, about 259.2 μL (0.25 mL) and 2592 μL (2.5 mL) is expected to be collected within 24 h. With 0.22 g of hydrogel and 20 mL saline, it takes about 4 h to reach an equilibrium condition. Thus, by using 1.32 g of hydrogel in the reservoir (6 times the higher magnitude weight of equivalent), it will not be an issue to store a 0.2 mL sample within 24 h. In order to optimize the geometry design of the reservoir, Fluent ANSYS is used to conduct fluid simulation analysis by varying the entrance quantity of the reservoir and the dimension of the diffuser. A diffuser is assigned as the reservoir entrance, and two air vents at both top-ends of the reservoir are assigned as outlets. The effect of the quantity of entrance ports and the width of the diffuser at the reservoir on the static area is simulated, and the results are as follows:

1. Effect of reservoir entrance quantity on the static area

With the increase of the inlet quantity from 1 to 3, the area whose velocity distribution is decreasing with the dead volume percentage increasing from 17.89% to 38.37% (static area) is shown in Figure 8B. Dead volume is the amount of storage below the minimum storage level that stays still or moves slowly. This means that these lower velocities will flow to the outlet, and a low dead volume percentage is preferable for reservoir design.

2. Effect of diffuser width, b on the static area

There is no significant difference in dead volume percentage with entrance increment width of 1.5 and 1.7 μm ($V_{d,\;1.5\mu m}$ = 17.89% and $V_{d,\;1.7\mu m}$ = 17.85%) as shown in Figure 8C. 

### 3.3. Characterization of Microfluidic Fabrication in the ESCARGOT Process

**Figure 9:**
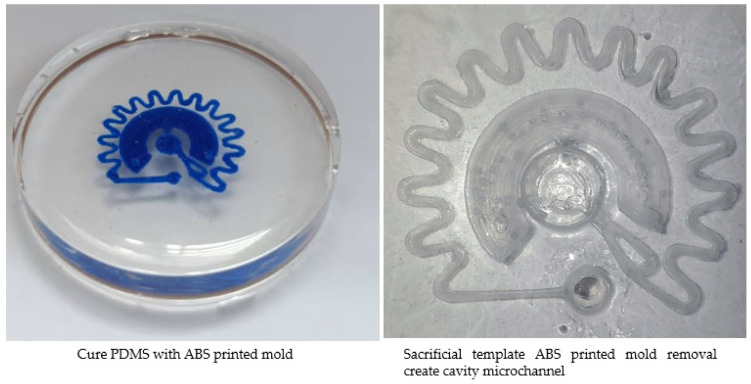
Schematic of the ABS printed mold-removal fabrication method for manufacturing microfluidic devices showed photo of cure PDMS with ABS printed mold and sacrificial template ABS printed mold removal create cavity microchannel.

Due to the limitations of the FDM machine, the proposed design necessitated support for printing and scaling up to 10 times in order to print the microchannel. Each batch of ABS-printed molds requires 15 min to be printed. The ABS-printed mold is immersed in PDMS, and the PDMS was cured at room temperature for 12 h. To create a cavity for a microfluidic device, the ABS-printed molds dissolved in cured PDMS using acetone. Templates mold complete removal is quite challenging especially for curved, wavy, and small microchannels [139]. Polyethylene glycol (PEG) was coated on the 3D-printed template to create a gap between the template and the PDMS after its removal [140]. It is easier to dissolve the template when there is a large gap between it and the PDMS. This alternative is a convenient method, especially when dissolving complex-shaped templates. To reduce the risk of clogging, the printed template is slowly dissolved from one point to another point. In addition, high pressures are often required when extracting a dissolved mold. The printed mold in the microchannel was not entirely eliminated from the PDMS microfluidic device during the initial dissolving step without sonication. They entirely removed using sonication [141]. To speed up the dissolution process, it is essential to ensure the circulation of fresh solvent solution [139]. Additionally, a transparent clear sample can be produced by the circulation of fresh solvent solution. Otherwise, it may turn a blueish color if immersed in an ABS-saturated acetone solution. Microchannels were successfully fabricated with complete ABS printed mold removal using the ESCARGOT techniques shown in Figure 9.

To investigate the effect of extrusion temperature, bed temperature, and printing speed on the structure and size of microchannels, the microchannel printed molds were printed at the extrusion temperature ranging from 230 to 250 °C with an interval of 5 °C, the bed temperature ranging from 80 to 100 °C with an interval of 5 °C, and the printing speeds ranging from 45 to 65 mm/min with an interval of 5 mm/min. While a critical key parameter is varied, other variables are kept constant, such as extrusion temperature was 230 °C, bed temperature was 80 °C, and print speed was 50 mm/s.

**Figure 10:**
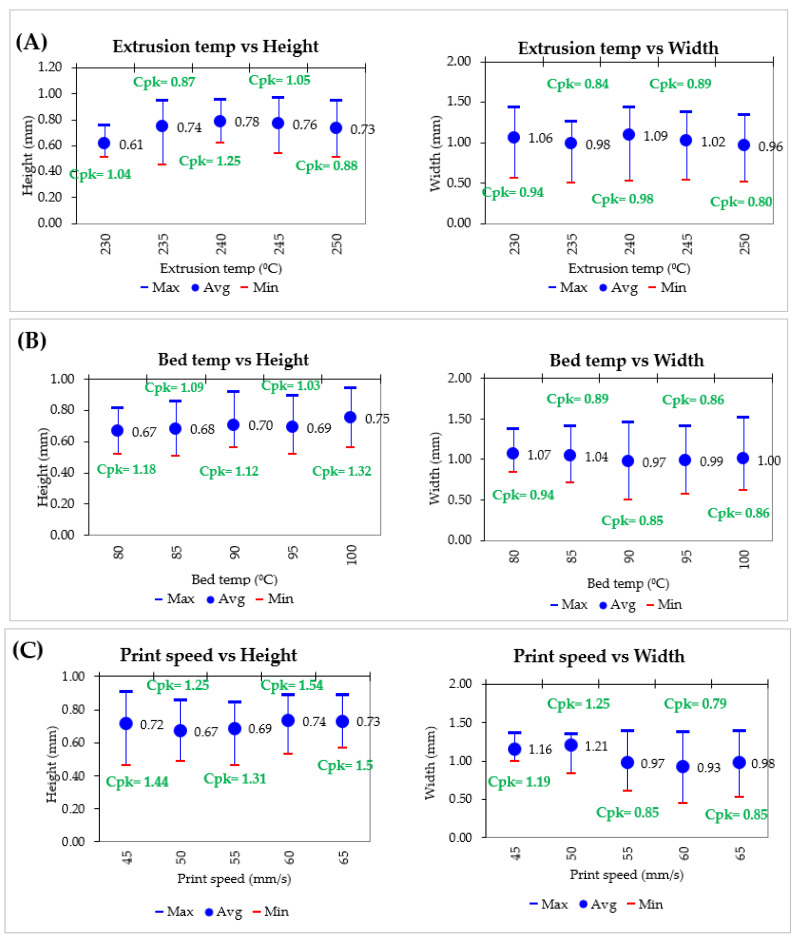
Height and width comparison in various extrusion temperature, bed temperature, and print speed.

The ABS printed mold is successfully printed, and there were no significant visual differences at the various extrusion temperatures, bed temperatures, and printing speeds. Figure 10 represents the comparison of height and width for an ABS printed mold when varying those parameters. For dimensional comparison, an extrusion temperature of 230 °C shown an average width near to the target value (0.5 mm). This is due to the fact that 230 °C is set closed to the ABS glass transition temperature (105 °C). Too high extrusion temperature compared to their glass transition temperature resulted in surface quality deterioration. At high temperatures, the viscosity is reduced, causing the fluid flow quickly and erratically, resulting in dimensional inaccuracies. When variable parameters are compared, there does not seem to be any difference in width and height of printed mold. The ability to release the thermal stress is reduced when the temperature differences between the extruder and the bed are large, leading to an increase in heat shrinkage and warpage. Dimensional accuracy suffers at high print speeds since there is less material extrusion and more time needed to connect a fresh layer in the molten state to an already-solidified layer. To print the ABS mold for the PDMS microchannel, the optimal condition parameters is chosen: 230 °C in extrusion temperature, 80–85 °C in bed temperature, and 45–50 mm/s in print speed. The tolerance for height is 0.5 ± 0.4 mm and for width is 1.0 ± 0.5 mm.

The replica moulding approach is used to effectively construct microchannels. The dimensions of the microchannel and the printed mold are quite significant. A printed ABS mold that provides dimensions for width and height results in good production. According to optical microscope observation, the PDMS microchannel surface was quite rough on the walls of the molds (~around few micrometers) [115], and this phenomenon also acknowledge by Rusli et al. [119]. Although the fluid flow would not be affected, the flow resistance would [123]. For the functional test, a flow rate ranging from 0.1 μL/min to 1 mL/min is used. It is observed that there is no sign of any blockages or leakage across the body of the microchannel. This demonstrates that the applied pressure induced in the microchannel is well within the PDMS matrix strength limit. The result supported by previous finding from Abidin et al. [117] and Rusli et al. [119] whereas high pressure inside the microchannel caused by the high flow rate leading to water leakage. Rusli et al. [119] demonstrate their model has no leakage when applied maximum volumetric flow rate of 9 mL/min. Rehmani et al. [115] also reported that a flow rate of 40 µL/min is achieved with no leakage in both straight and curved microchannels with a diameter of 0.4, 0.45, and 0.5 mm.

### 3.4. Experimental Versatility

**Figure 11:**
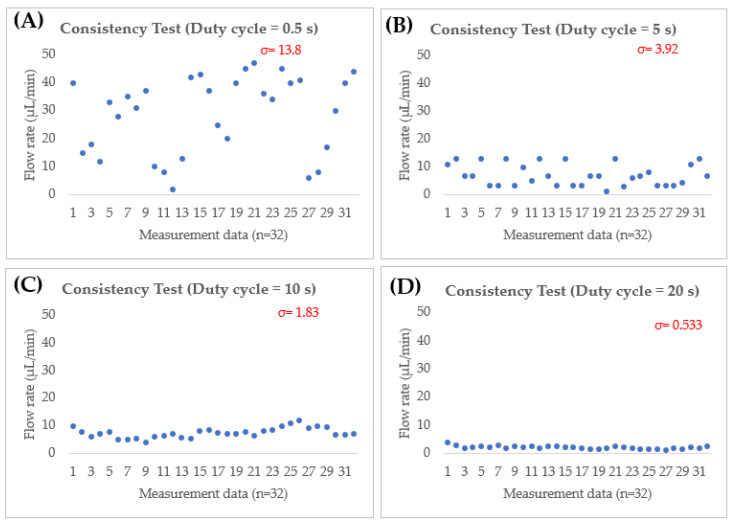
Flow rate for the operation of a finger actuated-based microfluidic system in (**A**) 0.5 s, (**B**) 5 s, (**C**) 10 s and (**D**) 20 s intervals.

For biomedical applications, the flow rate should be maintained at the desired flow rate for a certain period of time, especially in drug delivery and diagnostic system. The potential of the flow rate is not constant; it could be a result of losses caused by eddies or turbulence in the way of fluid flow in the microfluidic device itself, which could also be caused by bubbles. The resonant frequency in finger actuation can help to generate a constant and continuous flow. To demonstrate the consistency of the microfluidic system, we used repeated individual layer compressed and stretched procedures executed by a finger-actuated micropump with intervals of 0.5, 5, 10, and 20 s as per shown in Figure 11. Each data point represents the flow rate during the movement. Under a short duty cycle of 0.5 s, the average flow rate is 28.8 μL/min (v_max_ = 47 μL/min|v_min_ = 2 μL/min), shown the consistency is lower with large standard deviation (σ=13.82). This is due to the fact that it is difficult to develop consistent and fully deformation on the diaphragm. Thus, the output pressure became non-uniform under fast actuation [32]. The consistency improved by increase the duty cycle. Under duty cycle of 5 s, the average flow rate is 7.1 μL/min (v_max_ = 13 μL/min|v_min_ = 1.3 μL/min|σ=3.92). Under duty cycle of 10 s, the average flow rate is 7.6 μL/min (v_max_ = 12 μL/min|v_min_ = 4 μL/min|σ=1.84). Under duty cycle of 20 s, the average flow rate is 2.2 μL/min (v_max_ = 4.0 μL/min|v_min_ = 1.4 μL/min|σ=1.84). Based on this result, duty cycle of 20 s or more is the optimum condition for intended microfluidic system which posse’s lowest standard deviation.

Volumetric flow rate is defined as the amount of fluid moving per unit of time in cubic meters. As the prototype microfluidic device is scaled up 10 times higher from original design due to machine limitation, the actual obtained volumetric flow rate of $Q_{\exp(n=120)}=3.67\;\times \;10^{-11}{\;m}^{3}/s$ (2.2 μL/min under 20-s duty cycle actuation). Since the ISF and saline solution are compressible fluids, it would take ~67 s (about ~1 min) to completely fill up the cylindrical inlet (2.5 mm in diameter and 0.5 mm in height| V = 2.45 × 10^−9^ m^3^) and reach the sensor at the bottom of the inlet. The measured flow rate is 2.2 μL/min which is slightly higher than the scaled-up flow rate under ${*Q}_{\left(n=120\right)}=3.0\;\times {\;10}^{-11}{\;m}^{3}/s$ (1.8 μL/min), with an error gap of about 22%. Nonetheless, the measured flow rate value still meets the transdermal for medical application. In the prototype device, the volume of extracted saline is about 2 μL (thickness layer = 449 μm) available to be used for the sensor at one pressing. A single pressing generates a dimensionless length of 41.6 mm in the rectangular channel. Haldkar et al. [142] reported volumetric flow rate of 3.4 μL/s (240 μL/min) at their chamber and 0.471 μL/s (28.26 μL/min) available at biosensor location., which is higher than our prototype. This is due to the fact that our prototype placed the sensor at the bottom of the inlet, while theirs is placed 1 mm away from the MNs. It will be stored 3.16 mL with flow rate of 2.2 μL/min in the hydrogel reservoir within 24 h.

Weighted analysis is used to assess the diodicity of saline sample samples that are meant to represent ISF in order to analyze the prototype’s flow characteristics. Basically, backflow might occur when small volumes of flow crosstalk and asynchronous pumping is caused by a pressure difference at the inlet [31]. This combination design of Tesla-serpentine microchannel-hydrogel reservoirs demonstrates and proves that fluid can only flow in one direction. We also examined how long the prototype would last in operation and found that it could withstand 24 h of operation without leaking by manipulating them into cycles of pressing. Therefore, the result showed that our microfluidic flow-regulating device worked well within 24 h with no backflow. 

## 4. Conclusions

In a finger-actuated microfluidic system, an interstitial fluid (ISF) extraction component with a constant flow rate and no backflow is achieved by providing constant actuation displacement through chamber geometry with a duty cycle >20 s and integrating three components—a Tesla valve, a serpentine channel, and a hydrogel-assisted reservoir. We scaled up the prototype microfluidics device about ten times from the original design due to the limitations of 3D printing. Using an ISF extraction of 120 MNs, the flow rate discrepancy between the experimental and predicted flow rates is around 22%. It took about ~67 s to reach the sensor, which is located at the bottom of the inlet. In terms of consistency, the experimental result revealed that after 20 s of duty cycles with full deformation on the flexible diaphragm, the output pressure became uniform and the flow rate remained at nearly constant levels of 2.2 μL/min. It can be further optimized by increasing the duty cycle interval in order to achieve the desired target flow rate of 0.18 μL/min. This result demonstrates that finger actuation can be used to provide a constant flow rate. However, it is highly recommended that a miniaturized and compact structure of a piezoelectric micropump be integrated into the microfluidic device to enhance their functionalities into automated, fast mechanical response, no electromagnetic interference, and little noise. In terms of diodicity, when the serpentine microchannel and hydrogel-assisted reservoir are added to the microfluidic system integration, the diodicity increases by 2% and 34%, respectively, compared to when Tesla integration is used alone. Our prototype successfully maintains constant flow rate in liquid intake without any backflow (Di = 1.96). The hydrogel makes a significant contribution to the diodicity by directing fluid flow in one direction and stopping it from going in other directions, thus making it easier to control fluid flow. These significant flow characteristics demonstrate the potential usage in many low-cost and portable microfluidic applications. There are, however, several areas that still need significant improvement, especially in the low flow rate design of microfluidic devices, before the clinical transition can occur. First, maintaining a small but sufficient volume of analyte extraction for sensor detection within a certain period of time. Second, microfluidic flow dynamics are susceptible to disruption from bubble generation induced by high power sources or thermal energy; leakage, variation in pump volume stroke; gravitational; and motion flow effects; therefore, flow control is crucial and must be real-time and consistent in rate. Basically, for sensing detection, electron movement due to a chemical reaction on the working conductive electrode and a reduction reaction at another electrode is the basic mechanism by which glucose sensors measure current flow and convert to a measurable signal [143]. However, microfluidic work under low flow rates and small volumes is very challenging, especially at the micro- or nanoscale. High-sensitivity sensors will be needed to measure accurately at ultra-low flow rates and nano-volume sample volumes. To make sure the system works well, it is best to add a flow sensor that can measure the flow rate in a long-term, continuous, localizable, or multiple-point, fully automated way with minimal side effects on the normal flow pattern [144].

## Figures and Tables

**Table 1:** Geometry of design and prototype of microfluidic system.

Part	Parameter	Design	Prototype
Inlet	Inlet diameter, D_i_	0.25 mm	2.5 mm
	Inlet height, h_i_	0.05 mm	0.5 mm
Microchannel cross-sectional geometry	Width, w	0.10 mm	1.0 mm
	Height, h	0.05 mm	0.5 mm
Microchannel	Length, L(2→3)	0.84 mm	8.42 mm
	Length, L(3→4)	2.8 mm	28 mm
	Length, L(4→5)	0.3 mm	3 mm
Tesla	Length, L(5→6)	0.38 mm	3.8 mm
Pump chamber	Top diameter, D_p(T)_	0.5 mm	5 mm
	Top height, H_p(T)_	0.3 mm	3 mm
	Bottom diameter, D_p(B)_	0.3 mm	3 mm
	Bottom height, H_P(B)_	0.05 mm	0.5 mm
Diffuser	Width, a	0.05 mm	0.5 mm
	Width, b	0.15 mm	1.5 mm
	Length, L(8)	0.15 mm	1.5 mm
Reservoir	Outer Diameter, D_R1_	1.6 mm	16 mm
	Inner Diameter, D_R2_	0.8 mm	8 mm
Air vent	Diameter, D_o_	0.1 mm	1.0 mm

**Table 2:** Correlations of friction coefficients in rectangular channels.

First Author	Friction Coefficient Correlation	
Shah [125]	f=96Re1−1.3553β+1.9467β2−1.7012β3+0.9545β4−0.2537β5	(30)
Troniewski [124]	f=64Re* for laminar f=0.3164Re*0.25 where Re*=Re(2β)0.16	(31)
Kakac [126]	f=24Re1−1.3553β+1.9467β2−1.7012β3+0.9545β4−0.2537β5 For Smooth channel, aspect ratio β < 1	(32)
Spiga [127]	f=96Re1−1.20233β+ 0.88119β2+0.88819β3 −1.69812β4+0.72366β5	(33)
Hsieh [128]	f=48.1Re0.94(For Re<240)	(34)
Kandlikar [129]	f=0.0929+1.01612L/DeRe*−0.268−0.3293L/De where Re*=Re23+1124β(2−β	(35)
Zhai [130]	f=24Re1−1.3553β+1.9467β2−1.7012β3+0.9564β4−0.2537β5	(36)
Song [96]	f=0.8227Re0.42; Re ≤ 2700 f=0.0352Re0.0211; 2700 ≤ Re ≤ 4000 f=0.07019Re0.10498; Re ≥ 4000	(37)
